# Cardiac Dysphagia: When the Heart Disrupts Swallowing

**DOI:** 10.7759/cureus.109146

**Published:** 2026-05-18

**Authors:** Caitlyn Margol, Sajid Ali, Navkiran Randhawa, Subbaramiah Sridhar, Humberto Sifuentes

**Affiliations:** 1 Department of Gastroenterology and Hepatology, Augusta University Medical College of Georgia, Augusta, USA; 2 Department of Internal Medicine, Aiken Regional Medical Centers, Aiken, USA

**Keywords:** cardiac dysphagia, case series, endoscopy, esophageal compression, left atrial enlargement

## Abstract

Cardiac dysphagia is a rare cause of dysphagia due to an enlarged left atrium compressing the esophagus. We describe three patients (ages 70, 71, and 31) with past medical histories of advanced heart disease who presented with diverse clinical manifestations of dysphagia. Recognition of cardiac dysphagia is important to prevent unnecessary esophageal interventions. Multimodal evaluation with barium swallow, esophageal manometry, endoscopy, and cardiac imaging facilitates diagnosis and management. These cases highlight the importance of considering cardiac causes of dysphagia in patients with severe underlying heart disease.

## Introduction

Dysphagia is a common clinical complaint with diverse etiologies, broadly categorized into functional and mechanical causes. While gastroesophageal reflux disease (GERD), eosinophilic esophagitis, motility disorders (e.g., achalasia and diffuse esophageal spasm), and structural abnormalities (e.g., strictures and Schatzki rings) are well-recognized contributors, extrinsic esophageal compression remains an underappreciated cause. Extrinsic compression is typically associated with thoracic tumors or mediastinal masses. However, cardiovascular pathology, especially left atrial enlargement, can also cause dysphagia, termed "cardiac dysphagia" or "dysphagia megalatriensis" [1]. Due to the close anatomical relationship between the posterior left atrium and the mid-esophagus, significant atrial enlargement can compress the esophagus and lead to swallowing difficulties. Recognition of cardiac dysphagia is important, as misdiagnosis may lead to repeated or unnecessary expensive esophageal interventions, such as endoscopic dilations, that are not without complications.

Cardiac dysphagia is often overlooked because it can mimic more common esophageal disorders, resulting in delayed diagnosis and inappropriate management. Left atrial enlargement, often due to mitral valve disease, atrial fibrillation, or dilated cardiomyopathy, can cause progressive dysphagia [2]. Significant left atrial enlargement may also cause Ortner's syndrome (hoarseness due to left recurrent laryngeal nerve compression) or bronchial compression leading to lung collapse [3]. Overlapping symptoms with GERD and esophageal strictures may delay diagnosis. Advanced imaging, including barium esophagography, computed tomography (CT), and echocardiography, aids in differentiating cardiac dysphagia from other esophageal disorders and confirming the presence of cardiovascular compression [2]. Here, we present three cases of cardiac dysphagia to raise awareness of this clinical entity.

## Case presentation

Case 1

Case 1 is a 70-year-old man who presented with a two-month history of inability to tolerate oral intake, regurgitation of both solids and liquids, and choking on food. He had a six-pound weight loss over the prior month. A swallow study attempted by speech pathology was unsuccessful due to regurgitation of thin liquids. His symptoms were associated with dysphonia (hoarseness) that started around the same time as his lung cancer diagnosis eight months prior. At that time, a bronchoscopy was performed to sample lymphadenopathy, leading to a final staging of T3N2M1b lung adenocarcinoma. He did not have a mediastinoscopy. He also reported dyspnea on exertion after walking one block and noted that he had to breathe harder to speak. During an ear, nose, and throat (ENT) visit, a transnasal fiberoptic laryngoscopy with stroboscopy revealed bilateral true vocal fold (TVF) immobility in a partially abducted position exhibiting presbylaryngis.

The patient's past medical history includes congestive heart failure (CHF), atrial fibrillation, abdominal aortic aneurysm, and metastatic adenocarcinoma of the left lung, which spread to the pericardial sac and axilla. An electrocardiogram showed atrial fibrillation and left ventricular hypertrophy with a secondary repolarization abnormality (Figure 1). 

**Figure 1:**
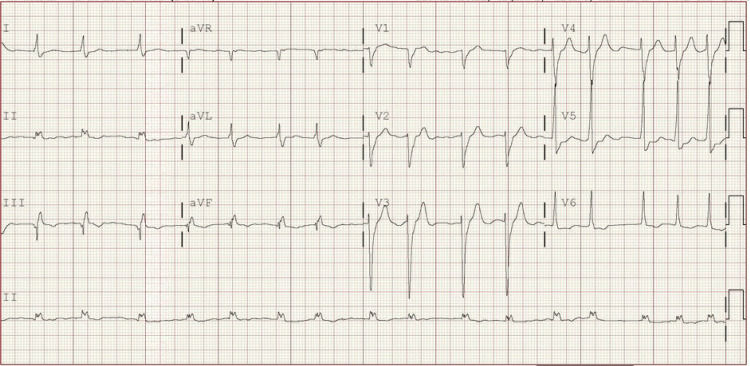
Electrocardiogram showing atrial fibrillation and left ventricular hypertrophy with a secondary repolarization abnormality

Multiple echocardiograms consistently showed severe left atrial dilation. The most recent echocardiogram performed prior to upper endoscopy indicated a left atrial volume index (LAVI) of 66 mL/m^2 ^(normal range: 19-41 mL/m^2^). During the endoscopy procedure, food chunks were noted in the mid/proximal esophagus (Figure 2A). There was difficulty traversing the adult endoscope beyond 30 cm. This area was serially dilated with a CRE balloon (8-9-10 and then 10-11-12); however, the endoscope was still unable to go beyond 30 cm. A pediatric scope was able to easily traverse the 30 cm mark and go through the gastroesophageal junction (Figure 2B), with a normal stomach seen (Figure 2C-2D). When reattempting passage with the adult endoscope, resistance was again encountered at 30 cm, with bulging from external compression most likely from his dilated left atrium (Figure 2E-2F). When attempting to push beyond 30 cm, the patient developed premature ventricular contractions, and the procedure was aborted. The patient's dysphagia was likely due to external compression from the dilated left atrium (cardiac dysphagia) noted at 30 cm, and because there were no intrinsic structural abnormalities of the esophagus leading to dysphagia, it was recommended that the patient receive percutaneous endoscopic gastrostomy (PEG) placement. 

**Figure 2:**
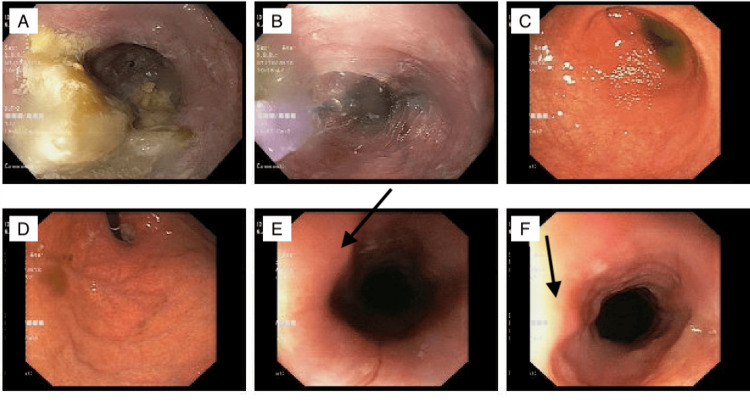
Endoscopy showing dysphagia secondary to compression from the dilated left atrium (cardiac dysphagia) (A) Retained chunks of food seen in the mid/proximal esophagus. (B) Squamocolumnar junction 40 cm from the incisors. (C-D) Normal stomach. (E-F) Dilated left atrium compression noted at 30 cm.

Case 2

Case 2 is a 71-year-old woman who presented with a chief complaint of excessive secretions in her throat, making her feel like she was choking. This was associated with nausea and a gagging sensation. She also reported continued reflux despite taking pantoprazole 40 mg twice daily and famotidine 20 mg once daily and endorsed some weight loss over the past year. She attributed the weight loss to not having much of an appetite and early satiety. She had a past history of dysphagia which necessitated six months of a PEG, removed three months prior to this current presentation. 

This patient's past medical history includes chronic GERD, hypertension, diabetes, hyperlipidemia, chronic obstructive pulmonary disease (COPD), coronary artery disease, history of coronary artery bypass graft surgery with multiple percutaneous coronary interventions, CHF, severe tricuspid and mitral regurgitation, and history of pulmonary embolism and right atrial thrombus (imaging suggests it was resolving with Eliquis). She also had an inferior vena cava (IVC) filter in place. An electrocardiogram showed atrial-sensed ventricular-pacing complexes (Figure 3). The most recent echocardiogram was remarkable for an LAVI of 62 mL/m^2^.

**Figure 3:**
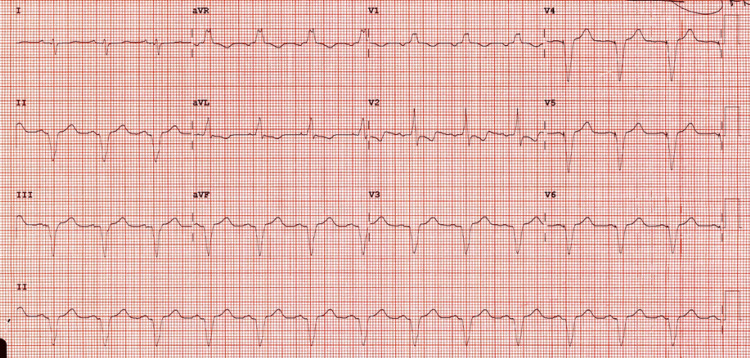
Electrocardiogram showing atrial-sensed ventricular-paced complexes

In light of her dysphagia eight months prior, a workup showed an abnormal esophageal manometry and impedance study. At about 32 cm from the incisors, there was an area with high pressure (extending about 4 cm) with an intervening low-pressure zone which could represent a diverticulum, non-obstructing mass, or ring/web, or external compression may be from cardiac indentation (Figure 4).

**Figure 4:**
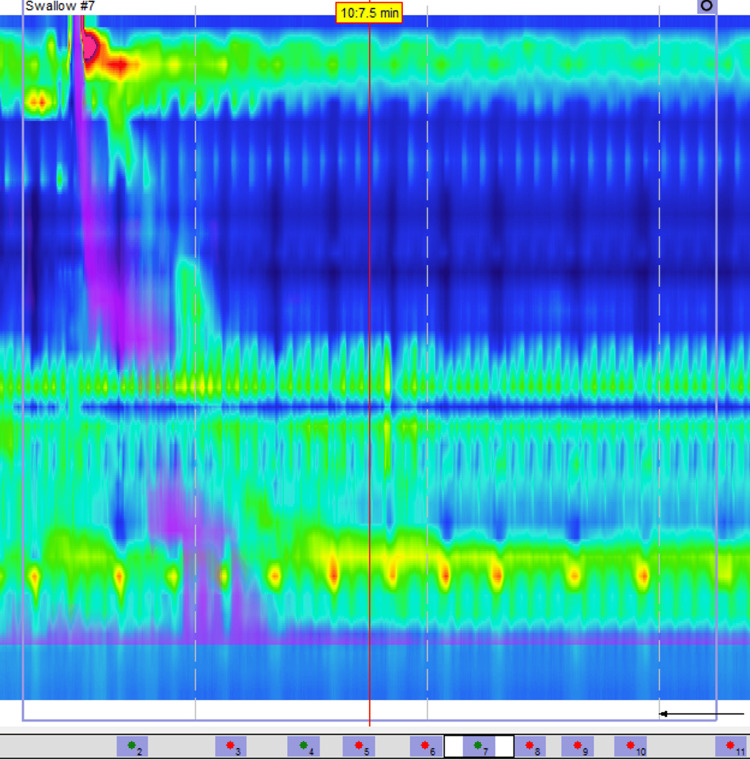
Esophageal manometry with impedance was abnormal. A 4 cm high-pressure zone was noted in the mid-esophagus with an intermittent pulsatile low-pressure pattern, perceived to be cardiac compression

Barium swallow prior to esophageal manometry showed evidence of esophageal dysmotility with tertiary contractions but no thoracic esophageal stricture. Extrinsic compression of the posterior cervical esophagus secondary to lower cervical spine anterior osteophytosis was noted. There was a delay in pharyngeal swallow noted as well as a very small type 1 hiatal hernia without gastroesophageal reflux. Interestingly, there was a subtle area of irregularity involving the right lateral cervicothoracic esophagus for which the radiologist suggested direct visualization via upper endoscopy would provide further evaluation for possible mucosal abnormality.

Six months after esophageal manometry, the patient underwent upper endoscopy for complaints of dysphagia. Endoscopy showed a normal esophagus, stomach, and duodenum (Figure 5A-5D). A prominent beating heart was seen at 32 cm with no obstruction (Figure 5E). Of note, several previous chest radiographs had shown the patient with stable cardiomegaly with an impression of an enlarged cardiopericardial silhouette with central pulmonary vascular congestion and mild interstitial edema, which is consistent with her increased fluid volume status. 

**Figure 5:**
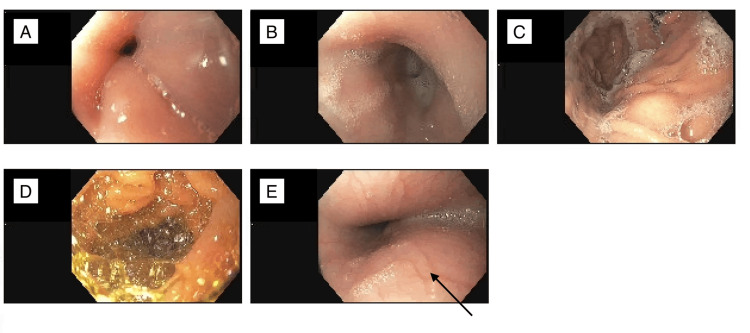
Endoscopy findings (A) No evidence of Barrett's, esophagitis, or esophageal web or ring. (B) Normal Z line and gastroesophageal junction at 37 cm. (C) Normal gastric body, fundus, antrum, and cardia. (D) Normal second and third parts of the duodenum. (E) Prominent cardiac indentation at 32 cm.

Case 3

Case 3 is a 31-year-old man who presented with progressive dysphagia to solids, intractable nausea and vomiting, constant epigastric pain, and ongoing bloating. His abdominal pain began about a year prior, with worsening of these symptoms over the past few months. He reported a sensation of food becoming stuck in his chest. His weight had been fairly stable, aside from losing 2-4 pounds the prior month. 

This patient's past medical history includes dilated cardiomyopathy, heart failure with reduced ejection fraction (HFrEF; EF <15%), atrial fibrillation, right middle cerebral artery (MCA) stroke without residual deficits, hypertension, and GERD. An electrocardiogram showed sinus tachycardia with atrial and ventricular premature complexes, as well as left atrial enlargement (Figure 6).

**Figure 6:**
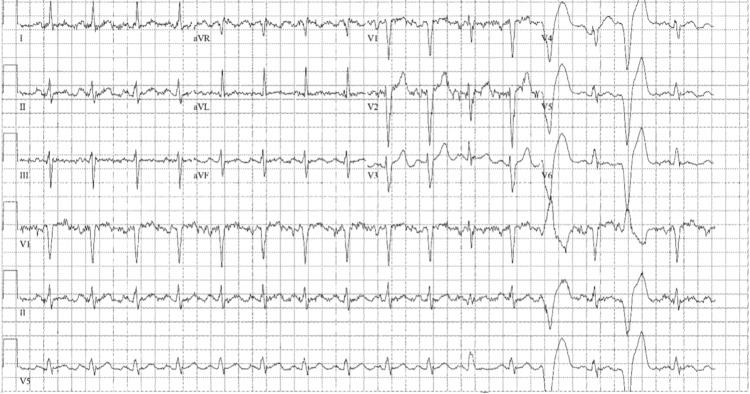
Electrocardiogram showing sinus tachycardia with atrial and ventricular premature complexes, as well as left atrial enlargement

Barium swallow showed normal esophageal motility, no evidence of hiatal hernia or spontaneous gastroesophageal reflux, and free passage of the 13 mm barium tablet through the esophagus into the stomach. Notably, the esophagus appeared narrowed due to the cardiac silhouette, with a prominent lumen reduction, suggesting cardiac dysphagia secondary to known cardiomyopathy (Figure 7). There was no evidence of laryngeal penetration, aspiration, esophageal web, or Zenker's diverticulum. The subsequent echocardiogram was remarkable for a severely dilated left atrium with an LAVI of 58.4 mL/m^2^. Two CT of the abdomen/pelvis scans performed in the prior two months were unremarkable for any intra-abdominal pathology.

**Figure 7:**
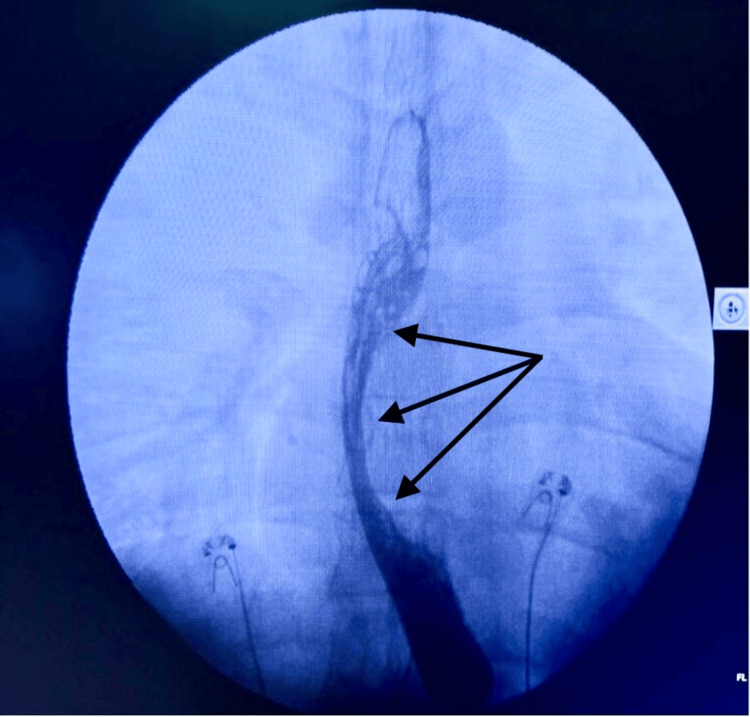
Barium swallow showing normal esophageal distension and motility with apparent narrowing due to cardiac silhouette

His nausea and vomiting improved with intravenous proton-pump inhibitor (IV PPI) and prochlorperazine. He was also given ondansetron and advised to follow up with gastroenterology outpatient with no further endoscopic evaluation performed inpatient.

Key clinical characteristics, diagnostic findings, and outcomes for all three cases are summarized in Table 1. 

**Table 1:** Comparison of the three cases LAVI: left atrial volume index; CHF: congestive heart failure; PEG: percutaneous endoscopic gastrostomy; CAD: coronary artery disease; HFrEF: heart failure with reduced ejection fraction; EF: ejection fraction; IV PPI: intravenous proton-pump inhibitor

Case	Age/sex	Cardiac diagnosis	LAVI (mL/m^2^)	Key diagnostic findings	Management
1	70 y/o M	CHF, atrial fibrillation, abdominal aortic aneurysm	66	Endoscopy showed external compression at 30 cm from the incisors; retained food in proximal esophagus	PEG placement after failed dilation and severe compressive symptoms
2	71 y/o F	CHF, severe mitral/tricuspid regurgitation, CAD	62	Endoscopy showed prominent cardiac indentation at 32 cm	Conservative management with fluid optimization and cardiac medications
3	31 y/o M	Dilated cardiomyopathy, HFrEF (EF <15%), atrial fibrillation	58.4	Barium swallow showed esophageal narrowing from cardiac silhouette	Conservative management with IV PPI and antiemetics

## Discussion

Cardiac dysphagia, particularly dysphagia megalatriensis, is a rare but clinically significant phenomenon characterized by esophageal compression due to cardiovascular abnormalities. The three cases presented here illustrate the diverse clinical manifestations and diagnostic challenges of this condition, each uniquely contributing to the growing body of literature on cardiac-induced dysphagia.

Pathophysiology and mechanism of dysphagia in cardiac conditions

Dysphagia secondary to cardiovascular causes arises from mechanical obstruction due to the anatomic proximity of the esophagus and the heart. The most commonly implicated structure is the left atrium, whose enlargement, typically due to chronic volume overload in conditions such as mitral valve disease, heart failure, and atrial fibrillation, can exert extrinsic compression on the esophagus [4]. This results in progressive dysphagia, often affecting solids initially before progressing to liquids.

In Case 1, a severely dilated left atrium with an indexed volume of 66 mL/m^2^ was noted, corresponding with external compression at 30 cm from the incisors, which led to a failed attempt at advancing an adult endoscope. In Case 3, a similarly dilated left atrium with an indexed volume of 58.4 mL/m^2^ resulted in prominent esophageal narrowing observed on barium swallow. These findings suggest that LAVI exceeding 55-60 mL/m^2^ may significantly increase the likelihood of symptomatic esophageal compression. In contrast, Deschepper et al. reported a case with a markedly higher LAVI of 143 mL/m^2^, further emphasizing the potential for severe esophageal involvement in cases of extreme atrial dilation [3]. However, it remains unclear whether a specific LAVI threshold should prompt transition from conservative management, such as diuresis and optimization of heart failure therapy, to invasive intervention including PEG placement or surgical correction. 

In Case 2, unlike the other cases, the dysphagia presented primarily as excessive throat secretions and gagging, rather than regurgitation or solid food obstruction. This case aligns with prior literature describing variations in dysphagia presentations, including globus sensation and esophageal dysmotility due to pulsatile cardiac structures [1]. 

Diagnostic approaches

The diagnosis of cardiac dysphagia remains challenging due to symptom overlap with primary esophageal motility disorders such as achalasia. A thorough evaluation includes barium swallow, esophageal manometry, endoscopy, and cardiac imaging. In all three cases, initial suspicion of structural esophageal pathology prompted further imaging; however, the identification of extrinsic compression emphasized the importance of integrating cardiac imaging into the diagnostic algorithm [4]. Esophageal manometry in Case 2 demonstrated a high-pressure zone at 32 cm, supporting prior reports of localized high-pressure areas due to external cardiac compression. Similarly, in Case 1, resistance to endoscopic passage at 30 cm further reinforced this finding, echoing previous cases where left atrial dilation induced segmental esophageal narrowing [5].

Management considerations

Management of cardiac dysphagia depends on the severity of symptoms and underlying cardiac pathology. While surgical intervention such as left atrial reduction or valve repair may provide definitive relief, it is often reserved for severe cases. Conservative approaches, including dietary modification, diuresis, and symptom control, are preferred in patients with advanced heart failure or poor surgical candidacy. In our case, the severity of compression and failed dilation necessitated PEG placement for nutritional support, a strategy previously reported in similar cases with refractory symptoms in nasogastric tube feeding [4]. In Case 3, treatment with IV PPI and antiemetics highlighted the role of medical management in cases without significant mechanical obstruction. Case 2 was managed conservatively with adjustments to fluid balance and cardiac medications, avoiding unnecessary invasive interventions.

Comparison with prior literature

The cases presented align with prior reports on dysphagia megalatriensis, particularly in patients with longstanding atrial fibrillation, valvular disease, or advanced heart failure [6]. Atrial sizes >8 cm are strongly associated with symptomatic esophageal compression, as seen in prior documented cases showing the importance of a high index of suspicion for cardiac dysphagia, particularly in patients presenting with unexplained dysphagia and known cardiomegaly [5]. Early recognition can prevent unnecessary esophageal interventions and guide appropriate cardiac management [4]. Cardiac dysphagia remains an underrecognized but clinically relevant entity, particularly in patients with significant left atrial enlargement. The cases presented illustrate the spectrum of disease severity, from mild symptoms managed conservatively to severe dysphagia requiring enteral feeding. A multidisciplinary approach incorporating gastroenterology, cardiology, and radiology is essential for accurate diagnosis and tailored management, including optimization of heart failure and thus volume status prior to endoscopic procedures such as esophageal dilation. Future studies should explore the role of novel imaging modalities and early cardiac interventions in mitigating esophageal compression and improving patient outcomes. Additional research is also needed to better characterize long-term clinical outcomes in patients with cardiac dysphagia.

## Conclusions

Dysphagia in patients with severe heart failure or cardiomyopathy may be an overlooked manifestation of cardiac dysfunction. Optimizing heart failure management can, in some cases, alleviate symptoms. Additionally, hoarseness and dysphagia in patients with concurrent lung cancer and cardiac disease should prompt consideration of cardiac compression of both the recurrent laryngeal nerve and esophagus. Given the significant overlap between cardiac dysphagia and primary gastroesophageal disorders, a high index of suspicion is essential to avoid unnecessary esophageal interventions. A multidisciplinary approach incorporating gastroenterology, cardiology, and radiology is crucial for accurate diagnosis and tailored treatment. Future studies should focus on advanced imaging modalities and interventional strategies to improve outcomes in patients with symptomatic esophageal compression. 

## References

[1] Yuan SM (2014). Cardiovascular dysphagia - anatomical and clinical implications. Folia Morphol (Warsz).

[2] Anduquia-Garay F, Santa-Gil V, Marquez-Parra NP, Cardona-Rios Y, Borraez-Segura B (2023). Cardiovascular dysphagia as a rare symptom of left atrial enlargement: a case report. Front Med Case Rep.

[3] Deschepper C, Devos D, DePauw M (2021). A case report: cardiac dysphagia-a ghost of the past?. Eur Heart J Case Rep.

[4] Nazeer NN, Anees CK, Paul A, Basheer A (2024). Dysphagia megalatriensis: a case report of potentially treatable etiology of dysphagia. Research Square.

[5] Miranda CJ, Palatnic L, Goyal S, Moyer RR, Nallapeta NS (2023). Dysphagia megalatriensis: a cardiac etiology masquerading as gastroesophageal dysphagia. Am J Gastroenterol.

[6] Mei A, Lachhar G, Ghayalod N, Mohsin S, Reilly J (2023). Dysphagia megalatriensis in a 93-year-old male with extensive cardiac history. Chest.

